# Pacific Ciguatoxin-1 (P-CTX-1) in a Moray eel (*Gymnothorax javanicus*) Responsible for Ciguatera in Khanh Hoa Province, Viet Nam

**DOI:** 10.3390/toxins17040186

**Published:** 2025-04-07

**Authors:** Ha Viet Dao, Hy Ho Khanh Le, Ky Xuan Pham, Vy Bao Phan, Anh Phuong Nguyen, Thiet Thi Doan, Xuan-Vy Nguyen, Nhu-Thuy Nhat Nguyen, Xuan-Thuy Thi Nguyen, Tung Ngoc Nguyen, Jiajun Wu, Jingyi Zhu, Leo Lai Chan

**Affiliations:** 1 Institute of Oceanography, Viet Nam Academy of Science and Technology (VAST), 01 Cau Da, Nha Trang 650000, Khanh Hoa, Vietnam; khighe2@yahoo.com (H.H.K.L.); kyjapan2004@yahoo.com (K.X.P.); baovyphan@gmail.com (V.B.P.); phuonganh.46cntp@gmail.com (A.P.N.); thamthietccb50@gmail.com (T.T.D.); nguyenxuanvi@gmail.com (X.-V.N.); nhat.thuy.174@gmail.com (N.-T.N.N.); xuanthuynguyen1293@gmail.com (X.-T.T.N.); 2 Center for High Technology Research and Development, Viet Nam Academy of Science and Technology. 18 Hoang Quoc Viet, Ha Noi 100000, Vietnam; tungnguyen@vast.vn; 3 State Key Laboratory of Marine Pollution and Department of Biomedical Sciences, University of Hong Kong, Hong Kong SAR 999077, China; jiajunwu@cityu.edu.hk (J.W.); jingyizhu9-c@my.cityu.edu.hk (J.Z.); leochan@cityu.edu.hk (L.L.C.)

**Keywords:** ciguatera, Pacific-ciguatoxin-1, moray, *Gymnothorax javanicus*, poisoning, Viet Nam

## Abstract

On 5 November 2020, a poisoning event involving four people by the consumption of moray eel occurred in Khanh Hoa Province, Viet Nam, with signs indicative of ciguatera. The remaining moray portion was confiscated for identification of causative species and responsible toxins. The phylogenetic study based on COX1 identified the moray as *Gymnothorax javanicus* Bleeker (1859). Out of 17 marine lipophilic toxins (MLPs) that were analyzed using LC-MS/MS, only Pacific ciguatoxin-1 (P-CTX-1) was detected in the moray’s flesh at 1.30 ± 0.004 ng/g ww, while no toxin was found in the skin. The N2a assay’s ciguatoxicities in the skin and flesh were 0.69 ± 0.075 and 2.49 ± 0.216 ng P-CTX-1/g ww equivalent, respectively. In the N2a assay, the P-CTX-1 amount in the moray flesh was 1.9 times greater than that determined by LC-MS/MS, indicating the presence of additional sodium channel activators or a matrix effect. The P-CTX-1 amount in the moray flesh was at a level that generates major ciguatera poisoning (CP) symptoms in humans (1.0 ng/g P-CTX-1), makes sense given that four consumers experienced the onset of poisoning symptoms. This study is significant for the management of seafood safety since it is the first scientific report on the species and toxin in a moray causing ciguatera in Viet Nam.

## 1. Introduction

Ciguatera poisoning (CP) causes gastrointestinal, neurological, and cardiovascular poisoning symptoms in humans who eat contaminated fish [1,2]. It is known that toxic benthic dinoflagellates, such as Gambierdiscus and Fukuyoa species, are the source of lipid-soluble polycyclic ether compounds called ciguatoxins (CTXs), which can accumulate in fish [3] and biomagnify along food chains [4]. As one of the top carnivorous predators, morays have been reported to collect larger levels of CTXs than other fish species [5,6,7]. Several tropical and subtropical fish species are known to possess CTXs. In some nations, including Japan, Hong Kong, and Kiribati, they are regarded as ciguateric fish [6,7,8,9]. 

In addition to two CP cases by the consumption of red snapper *Lutjanus bohar* in 2014 and 2016 that were documented [10], there are also suspected CP incidents involving the consumption of morays, mostly along the central coast of Viet Nam (Dao, unpublished data). In our previous investigation, it was found that four common moray species randomly collected from coastal locations where suspected CP cases have occurred had certain amounts of Pacific-ciguatoxin-1 (P-CTX-1), also referred to as ciguatoxin-1B (CTX-1B) [11]. In Viet Nam, it was proposed that these marine species might pose a health concern to humans. However, because there are not enough implicated samples, the toxins that cause these poisonings haven’t been confirmed. Furthermore, poisonous materials were typically cooked, processed, and partially consumed; as a result, it was unable to identify the causative species by morphological characteristics.

A poisoning event involving four people who ate a moray occurred in Khanh Hoa Province on 5 November 2020 (https://baokhanhhoa.vn/xa-hoi/yte-suckhoe/202011/bi-ngo-doc-nghi-do-an-ca-chinh-8192412/ (accessed on 8 November 2020)). This study presents the results of toxin analysis and species identification using a leftover piece of moray that was confiscated from this poisoning incident. Additionally, ciguatoxicity results in a mouse neuroblastoma (N2a) assay were documented. This study is significant for the management of seafood safety since it is the first scientific report on the causative species and responsible toxin in a moray that causes ciguatera in Viet Nam.

## 2. Results

### 2.1. Ciguatera Case

A 26-year-old man from Nha Trang city, Khanh Hoa Province, purchased a large moray (about 4.0 kg of body weight) at a local fish market. In order to share the moray with his relative family in Van Ninh District, Khanh Hoa Province, he split it in half. He kept part of the half to prepare his dinner at home. Following his meals, symptoms such as diarrhea, vomiting, and tingling in the limbs appeared; these were accompanied by ciguatera symptoms [12,13]. His health did not improve the next morning, so he was taken to Khanh Hoa General Hospital for medical treatment. Three of his family members who consumed the other half of the moray were also hospitalized after experiencing similar poisoning symptoms.

### 2.2. Species Identification

**Figure 1:**
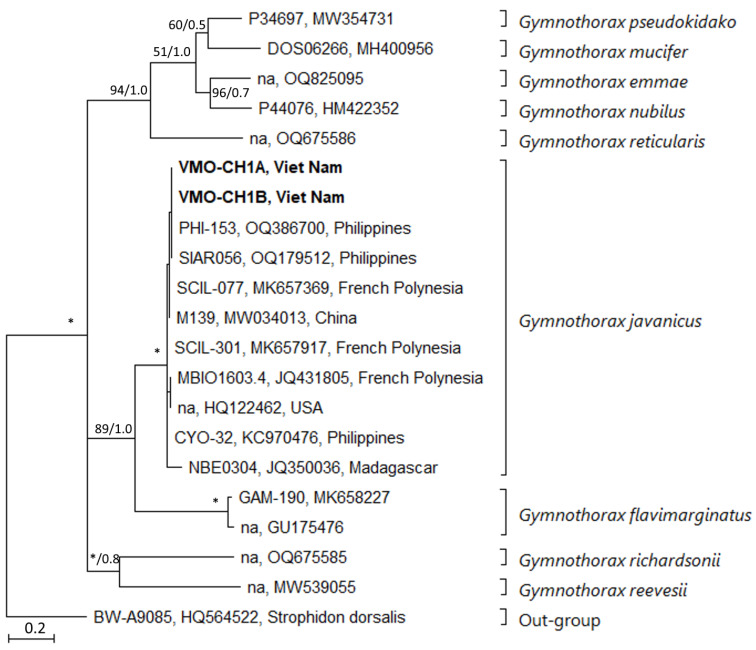
COX1 phylogeny of members of *Gymnothorax* inferred from Bayesian inference (BI) and maximum likelihood (ML). Samples collected from Viet Nam were in bold letters. *Strophidon dorsalis* was used as an out-group. Posterior probability and bootstrap values of each method are shown at each node: (**right**) BI and (**left**) ML; * denotes full support (ML = 100%, BI = 1.0).

The resulting COX1 alignment was 586 bp long and comprised 21 sequences, of which 155 (26.5%) were parsimony informative sites, 197 (33.6%) were variable sites, 43 (7.2%) were singletons, and 389 bp (66.4%) were conserved sites. The recently collected specimens from Viet Nam were placed into the clade composed of *Gymnothorax javanicus* Bleeker (1859) species with complete support, 100% and 1.0 bootstrap value and posterior probability, respectively, by phylogenetic analyses using ML and BI (Figure 1, Table A1). Sequence divergence values within the species *G. javanicus* varied from 0% (0 bp) to 2.2% (13 bp). Samples taken in the Philippines and Viet Nam do not differ in nucleotides. According to our COX1-based molecular analysis, the Vietnamese specimen is *G. javanicus*.

### 2.3. Toxin

**Figure 2:**
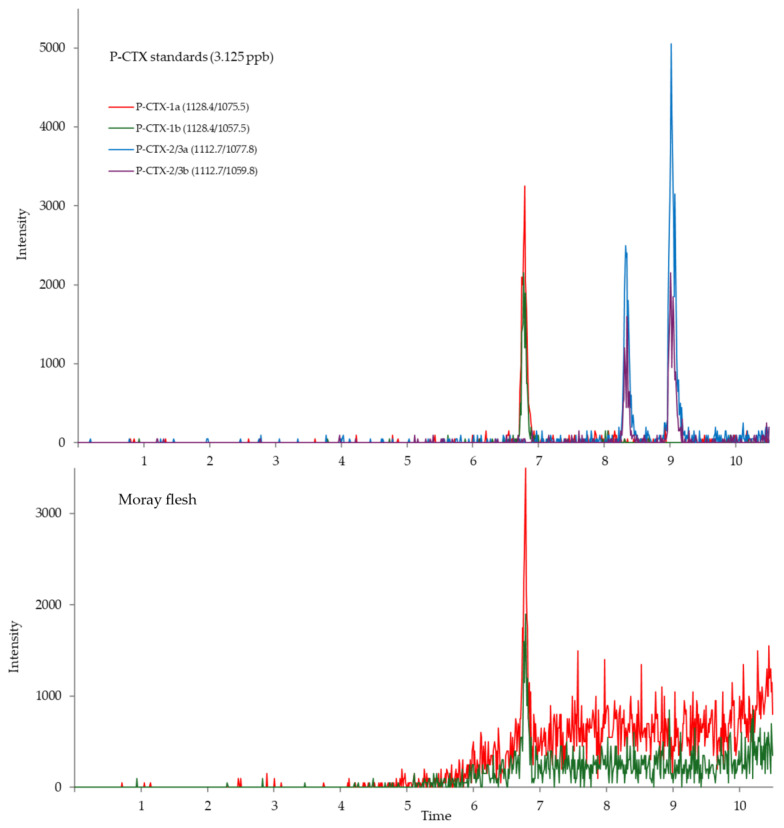
MRM LC-MS/MS extracted ion chromatograms of P-CTX standards and flesh of *Gymnothorax javanicus* specimen collected in the poisoning incident in Khanh Hoa Province, Viet Nam, in 2020 (P-CTX-1 detected at *m*/*z* 1128.4 > 1075.7, 1128.4 > 1057.5 and P-CTX-2/3 detected at *m*/*z* 1112.7 > 1077.8 and 1112.7 > 1059.8 for 3.125 ppb).

Among the 17 marine lipophilic phytotoxins (MLPs), only P-CTX-1 was detected in the moray flesh sample at 1.30 ± 0.004 ng/g ww by setting the multiple reaction monitoring (MRM) transitions as *m*/*z* 1128.4 > 1057.5, 1128.4 > 1075.5, and 1128.4 > 1093.5. Nevertheless, the skin sample did not contain any toxins. The MRM LC-MS/MS chromatogram of the P-CTX standards and flesh sample from *G. javanicus* are shown in Figure 2.

The ciguatoxicities in the skin and meat samples were determined in the N2a assay to be 0.69 ± 0.075 and 2.49 ± 0.216 ng P-CTX-1/g ww equivalent, respectively (Table 1). 

## 3. Discussion

The results show that P-CTX-1 is dominant in Viet Nam, which is consistent with our earlier findings on the toxin in the red snapper *Lutjanus bohar* [10] and some moray species [11] found in Vietnamese coastal waters. The accumulation of CTXs in fish originating from toxic benthic dinoflagellates is well documented [6,14]. A number of possible CTX-producing dinoflagellates, such as *Gambierdiscus* species, were reported in Viet Nam [15] and have since been updated [16]. To better understand the mechanism of the CTXs pathway in marine ecosystems, research is being performed on the presence of CTXs in morays with the distribution of toxic dinoflagellates, *Gambierdiscus* spp. 

The P-CTX-1 amount of 1.30 ± 0.004 ng/g found in the flesh of *G. javanicus* in the present study was lower than the 4.07 ng/g found in a specimen of this species from Spratly Islands in 2018 [11]. Only P-CTX-1 was detected by LC-MS/MS in our study, even though numerous P-CTXs were found in different samples. According to an earlier study, P-CTX-2 is the main P-CTX in herbivorous and omnivorous fish, while P-CTX-1 is the dominating P-CTX in top predators like moray eels [17]. Given their perhaps lower quantities in the sample and the strong matrix effects that can obstruct the detection procedure, the absence of P-CTX-2/3 in the chromatogram may be explained.

According to Chan et al. [6], ciguatoxicity in morays can vary up to 10,000 times between species and localities. Additionally, it was discovered that, although it was still unclear for many species, the concentration of CTXs correlated well with the moray’s body weight [6,18]. Due to the limitation of sample size and sampling sites, more work on CTX research is required to obtain a comprehensive picture of the prevalence of ciguatera in Viet Nam. The amount of P-CTX-1 in this study was beyond the EFSA-recommended level of 10 pg/g P-CTX-1 equivalent, even at a dose that induces notable CP symptoms in people [4]. The outcome makes sense given that four people who consumed this moray experienced the onset of poisoning symptoms. 

The moray, *G. javanicus*, is known to accumulate high CTX levels, and with their large body sizes, they have the potential to cause mass ciguatera poisoning and even deaths in several countries [2,4,19,20,21,22]. This species’ extensive range has led to a number of incidents in the Atlantic and Indo-Pacific ocean regions [23]. In Viet Nam, *G. javanicus* is a common moray species [24,25] that is frequently consumed by both locals and foreign visitors. Together with our previous result [11], this one warns that moray *G. javanicus* represents a ciguatera threat in Viet Nam. In order to protect human health, there should be a stronger public awareness campaign and updated regulations on the consumption of this marine species in Viet Nam. 

Complex biological matrices can affect measurement accuracy and dependability, frequently producing false-negative results in instrumental analysis and false-positive results in bioactivity analysis. In the present study, the corresponding P-CTX-1 level in the flesh sample of *G. javanicus* in the N2a assay was 1.9 times greater than the LC-MS/MS measurement. This outcome is in line with the previous study [26]. This discrepancy could be caused by matrix effects in the sample, additional sodium channel activators, or unknown toxins. Instrumental methods are unable to quantify these unknown toxins. 

The residual fish oil content is probably the cause of matrix effects in the LC-MS/MS detection of CTXs. For instance, compared to pure methanol, fish flesh extracts can reduce the P-CTX-1 standard response by 34–40% [26]. Likewise, compared to fish flesh extracts, matrix effects were much diminished in fish blood extracts [27]. These suggested that one of the key causes of matrix effects is the residual fish oil concentration. The skin sample from the current study showed clear matrix interference, and the high lipid content in the LC-MS/MS analysis was blamed for signal suppression. Improvements in clean-up procedures for CTX detection are necessary in Viet Nam in order to manage fisheries resources for CFP control and assess threats to human health. 

Since CTXs preferentially accumulated in fatty tissues, most recent investigations on their content concentrated on the fish’s meat and viscera. Limited research has been performed on other fish tissues. Using mouse bioassay, it was discovered that ciguatoxin is present in the gonads, gills, heart, skin, blood, flesh, and bones of Caribbean fish, and that the toxin concentration in the flesh was comparable to that in the skin [28]. Using the N2a assay, Roué et al. [29] discovered that the ciguatoxicity in gigantic clams occurred in the following order: viscera > flesh > mantle. In the present study, the skin of *G. javanicus* has a lower level of N2a ciguatoxicity than the flesh. More research is necessary to understand the relationship between the ciguatoxicity of this species’ flesh and skin because of the small sample size. Additionally, more research is needed to determine the tissue distribution of ciguatoxicity in the moray *G. javanicus* for application in public health and fisheries management.

## 4. Materials and Methods

### 4.1. Sample Collection

**Figure A1:**
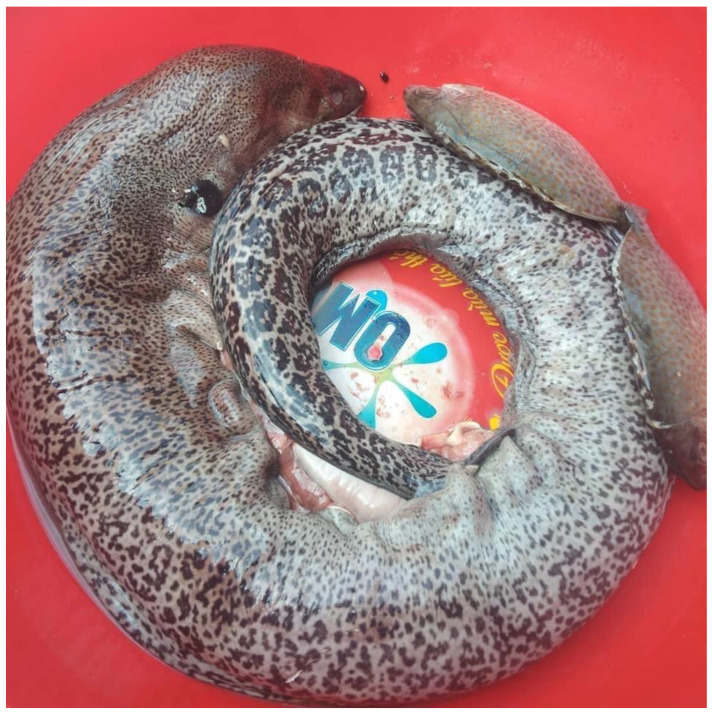
Moray specimen that caused the poisoning incident in Khanh Hoa Province, Viet Nam, in 2020 (photo was provided by the victim’s family).

A portion of moray (Figure A1) was taken from the victim’s residence in Nha Trang city, Khanh Hoa province, and brought to the laboratory in a cool state. In order to identify the species using molecular techniques, approximately 5 g of wet weight (ww) flesh was gathered. The remaining moray sample was divided into sub-samples of flesh (296 g) and skin (110 g). These subsamples were subsequently stored in the refrigerator (2–10 °C) for toxin analysis after being lyophilized to the final 89 g and 68 g of freeze-dried materials, respectively. 

### 4.2. Reagents and Chemicals

DNeasy^®^ Blood & Tissue were commercial products sourced from Qiagen (Hilden, Germany); acetate and EDTA were sourced from Sigma-Aldrich (Hamburg, Germany); Midori Green Advance was sourced from Nippon Genetics Europe GmbH (Düren, Germany); GenElute™. PCRClean-Up kit was sourced from SigmaAldrich (St. Louis, MN, USA); Tris base was sourced from Merck (Frankfurt, Germany); GeneRuler DNA Ladders were sourced from Thermo Fisher-Scientific (Bremen, Germany).

A total of 17 typical marine lipophilic phycotoxin (MLP) standards (azaspiracids 1–3; brevetoxins-2, -3, and -9; gymnodimine; spirolide 1; okadaic acid; dinophysistoxins 1–2; pectenotoxin 2; yessotoxins (YTX) and the YTX derivative homoYTX; P-CTX-1; P-CTX-2; and P-CTX-3) were mixed and diluted for constructing a calibration curve with 80% aqueous methanol. Milli-Q water from a Milli-Q water-purification system (Millipore, Billerica, MA, USA) was used throughout the experiment. HPLC-grade methanol and acetonitrile were purchased from Merck (Darmstadt, Germany). Pesticide-grade n-hexane was purchased from Anaqua Chemicals Supply (Wilmington, NC, USA). Acetic acid (99.9%) was purchased from Wako Pure Chemical Industries (Osaka, Japan). AR-grade chloroform was obtained from LabScan (Bangkok, Thailand). AR-grade formic acid (98–100%), ammonia formate (98–100%), and ammonia solution (25%) were supplied from Sigma-Aldrich (St. Louis, MO, USA).

### 4.3. Species Identification

As mentioned above, since the poisonous sample could not be identifiable by morphological characters, molecular techniques were applied in this study for species identification.

Following the manufacturer’s instructions, 100 mg of flesh tissue from two different parts was extracted using the DNeasy Blood & Tissue Kit, Qiagen (Germantown, MD, USA). The regions selected for PCR amplification were portions of the cytochrome oxidase subunit 1 (COX1). We used the primer pairs of FishF1/FishR1 [30] to obtain the length of 650 bp under the PCR amplification protocols of Weigt et al. [31]. The GenElute^TM^ PCR Clean-Up kit (SigmaAldrich, St. Louis, MI, USA) was used to clean PCR products. 1ST BASE (Selangor, Malaysia) conducted direct Sanger sequencing of PCR products in both directions. The consensus sequences were assembled in Clone Manager 9 (Sci-Ed, Cary, NC, USA). Alignments were cut at the 5′ and 3′ ends prior to divergence analysis in order to remove missing data. The internet program Blast, located at https://blast.ncbi.nlm.nih.gov/Blast.cgi (accessed on 15 March 2024), was used to compare the consensus sequences determined in this work to GenBank. 

### 4.4. Phylogenetics

Two consensus sequences obtained in this study were compared to COX1 sequences, eighteen sequences of known *Gymnothorax* species, and one sequence from members *Strophidon* (out-group) retrieved from GenBank (Table A1) for forming the final COI alignment. The datasets were aligned by the MAFFT algorithm with the selection of the q-insi option. jModelTest v.2.1.6 [32] and the corrected AIC (Akaike information criterion) were used to find the best evolutionary model for each alignment. The phylogenetic analyses were carried out using Bayesian inference (BI) and maximum likelihood (ML). A General Time Reversible model and 1000 bootstrap replicates were used to perform ML using RAxML v.8.1 [29]. MrBayes v.3.2.2 [30] was used to conduct BI analyses, utilizing the same model as before. Two parallel runs with four chains each (three heated and one cold) were carried out in the BI, sampling a tree every 100 generations.

### 4.5. Toxin Extraction and Clean-Up

Targeted MLPs in moray samples were extracted and cleaned up using the procedures outlined by Zhu et al. [17]. Briefly, 1 g of freeze-dried flesh (corresponding to 3.32 g ww) and 0.5 g of freeze-dried skin (corresponding to 0.81 g ww) were mixed with diatomaceous earth and extracted using the ASE 200 system (Dionex, Sunnyvale, CA, USA). The extract was concentrated and then dissolved in 25% aqueous methanol. Each crude extract was loaded on a C18 SPE cartridge (Agilent BondElut, Santa Clara, CA, USA; 500 mg, 6 mL) and the analytes were eluted by 4 mL of methanol and 4 mL of methanol containing 0.3% ammonia solution. The eluent was washed with n-hexane and further extracted with chloroform. The chloroform layer was concentrated and dissolved in 5% methanol in chloroform. The analyte was passed through a silica cartridge (Waters Sep-Pak, Milford, MA, USA; 500 mg, 6 mL). All the target analytes, excluding YTX, were eluted using 10% methanol in chloroform. YTX was then eluted using 8 mL of 30% methanol in chloroform. The eluent was dried under N_2_ and was resuspended with 100 μL of 80% methanol/water before LC-MS/MS injection.

### 4.6. LC-MS/MS Analysis

MLP concentrations were determined by an Agilent 1290 Infinity ultra-performance liquid chromatograph (UPLC) (Agilent, Palo Alto, CA, USA) interfaced with a 5500 QTRAP system (AB Sciex, Foster City, CA, USA) in both positive and negative ions. All the target analytes were detected in multiple-reaction-monitoring mode. Quantitation and confirmation mass transitions of each MLP showed in Table 2. A Phenomenex Kinetex C18 LC column (100 × 2.1 mm i.d., particle size 1.7 μm) was used for the separation. The injection volume was 5 μL. The electrospray ionization parameters were set as follows: positive ion-spray voltage, 5500 V; negative ion-spray voltage, −4500 V; GS1, 30 psi; GS2, 40 psi; curtain gas, 10 psi; ion source temperature, 400 °C; collision gas, medium. Gradient elution was performed at a flow rate of 0.2 mL/min with (A) Milli-Q water containing 0.1% formic acid and 2 mM ammonium formate and (B) 95% acetonitrile in Milli-Q water containing 0.1% formic acid and 2 mM ammonium formate. The gradient program started at 20% B and was maintained for 1 min. It was then increased to 80% B in 5 min and maintained for 2 min. After increasing to 100% B in 2 min and maintaining it for 4 min, it then returned to 20% B in 0.1 min. The column was equilibrated with 20% B for 4.9 min before the next run. The quality assurance of the method was assessed by spiking 0.1 ng MLPs into 0.5 g of flesh (dry weight) of nontoxic mangrove red snappers purchased from the market (n = 3). The limit of detection (LOD) and limit of quantification (LOQ) were defined based on an instrumental signal-to-noise ratio of 3:1 and 10:1, respectively. In this method, the LOD of P-CTX-1 was 0.078 ng/g, and the LOQ was 0.31 ng/g. 

### 4.7. Mouse Neuroblastoma (N2a) Assay

The ciguatoxicities of moray flesh and skin samples were also determined by the N2a assay [26]. The final extracts for LC-MS/MS analysis were diluted five times using 20% methanol in PBS and then applied to N2a cells. An N2a cell cytotoxicity assay was carried out using previously published techniques [6,33]. N2a cells (CCL131; ATCC, Manassas, VA, USA) were cultured in RPMI-1640 medium (Gibco, Life Technologies, Carlsbad, CA, USA) with 10% fetal bovine serum (BD Biosciences, San Jose, CA, USA) and 2 g/L of Na_2_CO_3_ at 37 °C in 5% CO_2_. N2a cells were seeded into 96-well culture plates at a cell density of 2.5 × 10^5^ cells/mL with 200 μL of the medium. After incubating for 24 h, the medium was renewed with complete RPMI-1640 (including 0.1 mM ouabain and 0.01 mM veratridine). A total of 10 µL/well P-CTX-1 standards were added to the cells at 08 concentrations ranging from 4.88 pg/mL to 312 pg/mL in 06 replicates. Sample extracts dissolved in 20% methanol in PBS were diluted and tested in triplicate. After incubating for 18 h, the cell proliferation was measured by MTT (3-(4,5-dimethylthiazol-2-yl)-2,5-diphenyltetrazolium). A microplate reader with a reference wavelength of 655 nm was used to measure absorbance at 595 nm. The optical density obtained from each well was normalized by MTT blank. Ciguatoxicity values of the moray samples were determined from the standard curve. The assay was performed twice, and the ciguatoxicity values are reported as mean P-CTX-1 equivalents between the two assays. The LOQ of this assay was 9.75 pg/g ww.

## Figures and Tables

**Table 1:** The ciguatoxicities determined in N2a assay of *Gymnothorax javanicus* specimen collected from the poisoning incident in Khanh Hoa Province, Viet Nam, in 2020.

Sample	Dry Weight (g)	Wet Weight (g)	Toxicity (pg/g ww P-CTX-1 eq.)
Flesh	1	3.32	2.49 ± 0.216
Skin	0.5	0.81	0.69 ± 0.075

**Table 2:** Mass transitions for the 17 MLPs.

Toxin ID	Quantitation Mass Transitions (*m*/*z*)	Confirmation Mass Transitions (*m*/*z*)
P-CTX-1	1128.4 > 1075.5	1128.4 > 1093.5 1128.4 > 1057.5
P-CTX-2	1112.7 > 1077.8	1112.7 > 1059.8 1112.7 > 1041.8
P-CTX-3	1112.7 > 1077.8	1112.7 > 1059.8 1112.7 > 1041.8
Azaspiracid-1	842.5 > 806.4	842.5 > 824.6
Azaspiracid-2	856.4 > 820.6	856.4 > 838.6
Azaspiracid-3	828.4 > 792.5	828.4 > 810.5
Gymnodimine	508.4 > 120.2	508.4 > 490.3
Spirolide-1	692.4 > 444.4	692.4 > 674.3
Pectenotoxin-2	876.5 > 823.5	876.5 > 805.5
Brevetoxin-2	912.4 > 895.4	912.4 > 877.4
Brevetoxin-3	897.5 > 725.5	897.5 > 879.7
Brevetoxin-9	899.6 > 881.5	899.6 > 863.6
Okadaic acid	803.6 > 255.0	803.6 > 113.1
Dinophysistoxin-1	817.4 > 255.0	817.4 > 113.0
Dinophysistoxin-2	803.6 > 255.0	803.6 > 113.1
Yessotoxin	1141.5 > 855.2	1141.5 > 713.2
Homo yessotoxin	1155.6 > 1075.6	1155.6 > 869.6

## Data Availability

The original contributions presented in this study are included in the article. Further inquiries can be directed at the corresponding author(s).

## Appendix A

**Table A1:** List of taxa and GenBank accession numbers of *Gymnothorax* spp. used in this present study. Samples produced in this study are written in bold; na, = not available; -/-, as above.

Taxa	Voucher Number	Locality	GenBank Accessions	References
*Gymnothorax emmae*	na	China	OQ825095	Direct submission
*Gymnothorax flavimarginatus*	na	na	GU175476	[34]
*Gymnothorax flavimarginatus*	GAM-190	French Polynesia	MK658227	[35]
***Gymnothorax javanicus***	**VMO-CH1A**	**Viet Nam**		**This present study**
***Gymnothorax javanicus***	**VMO-CH1B**	**Viet Nam**		**This present study**
*Gymnothorax javanicus*	PHI-153	Philippines	OQ386700	Direct submission
*Gymnothorax javanicus*	SIAR056	Philippines	OQ179512	-/-
*Gymnothorax javanicus*	CYO-32	Philippines	KC970476	-/-
*Gymnothorax javanicus*	M139	China	MW034013	-/-
*Gymnothorax javanicus*	SCIL077	French Polynesia	MK657369	[35]
*Gymnothorax javanicus*	SCIL301	French Polynesia	MK657917	-/-
*Gymnothorax javanicus*	MBIO1603.4	French Polynesia	JQ431805	[36]
*Gymnothorax javanicus*	na	USA	HQ122462	Direct submission
*Gymnothorax javanicus*	NBE0304	Madagascar	JQ350036	[36]
*Gymnothorax mucifer*	DOS06266	Taiwan	MH400956	[37]
*Gymnothorax nubilus*	P44076	New Zealand	HM422352	Direct submission
*Gymnothorax pseudokidako*	NMMB-P34697	Taiwan	MW354731	[38]
*Gymnothorax reevesii*	na	China	MW539055	Direct submission
*Gymnothorax reticularis*	na	China	OQ675586	-/-
*Gymnothorax richardsonii*	na	China	OQ675585	-/-
*Strophidon dorsalis*	BW-A9085	Indonesia	HQ564522	-/-
